# Real-time million-synapse simulation of rat barrel cortex

**DOI:** 10.3389/fnins.2014.00131

**Published:** 2014-05-30

**Authors:** Thomas Sharp, Rasmus Petersen, Steve Furber

**Affiliations:** ^1^School of Computer Science, The University of ManchesterManchester, UK; ^2^Laboratory for Neural Circuit Theory, RIKEN Brain Science Institute, WakoshiSaitama, Japan; ^3^Faculty of Life Sciences, The University of ManchesterManchester, UK

**Keywords:** SpiNNaker, simulation, barrel, large scale, real time

## Abstract

Simulations of neural circuits are bounded in scale and speed by available computing resources, and particularly by the differences in parallelism and communication patterns between the brain and high-performance computers. SpiNNaker is a computer architecture designed to address this problem by emulating the structure and function of neural tissue, using very many low-power processors and an interprocessor communication mechanism inspired by axonal arbors. Here we demonstrate that thousand-processor SpiNNaker prototypes can simulate models of the rodent barrel system comprising 50,000 neurons and 50 million synapses. We use the PyNN library to specify models, and the intrinsic features of Python to control experimental procedures and analysis. The models reproduce known thalamocortical response transformations, exhibit known, balanced dynamics of excitation and inhibition, and show a spatiotemporal spread of activity though the superficial cortical layers. These demonstrations are a significant step toward tractable simulations of entire cortical areas on the million-processor SpiNNaker machines in development.

## 1. Introduction

The rodent somatosensory cortex is principally concerned with processing information from the whiskers, and is organized accordingly (Petersen et al., 2009). In common with the other sensory cortices, the barrel cortex is radially organized into granular (layer 4), supragranular (layers 1–3) and infragranular layers (layers 5, 6). In lateral organization, it contains a topographic map of the animal's snout, in that layer 4 consists of discrete barrels that can be visualized by cytochrome oxidase staining. A barrel column is defined as a cylinder through all cortical layers, with a cross-sectional area equal to that of the granular-layer barrel. Each whisker is represented primarily by a corresponding barrel column, and the spatial arrangement of the barrel columns reflects that of the whiskers (Petersen, 2007). Axonal projections from the ventral posteromedial nucleus of the thalamus, which convey sensory signals from the whiskers, primarily innervate the granular layer. Broadly, signals flow within a barrel column from granular to supragranular layers and in turn to infragranular layers (Lefort et al., 2009). This relatively clear and well understood organization makes the barrel cortex a good candidate for investigations of cortical microcircuitry.

Pioneering models of the rodent whisker barrel reproduced the thalamocortical response transformations observed by Simons and Carvell (1989) but used very few neurons because of limitations in the computing resources available at the time of publication (Kyriazi and Simons, 1993) or represented whole-population activity as a single firing-rate state variable in order to analyze the dynamics of the network (Pinto et al., 2003). In making such abstractions, these models, respectively, may have introduced artefactual finite-size effects and failed to demonstrate the mechanisms by which discrete spikes process information *in vivo*.

Ever larger and more detailed models are becoming feasible as high-performance computing enjoys an exponential growth in power. However, structural and functional disparities remain between organic and silicon computers that limit the scale of neural-circuit simulations. High-performance computers comprise some thousands of processors and, typically, use point-to-point communication channels and global synchronization mechanisms. In contrast, the brain uses billions of processing units that communicate across intricate axonal and dendritic trees and synchronize, if at all, through decentralized, recurrent feedback loops. Analog computer architectures, such as BrainScaleS (Schemmel et al., 2010), partially bridge this gap by directly modeling membrane-potential dynamics with very few transistors in subthreshold states, but these architectures still face problems of communication and raise entirely new challenges of configuration and programming. Graphics processing units are also commonly used in neural-circuit simulations, but suffer from significant energy requirements and communications bottlenecks.

SpiNNaker is designed to emulate the structure and function of neural tissue using very many low-power digital processors and an interprocessor communication mechanism inspired by axonal arbors. Here, we demonstrate that prototype SpiNNaker hardware comprising one thousand processors is able to simulate a model of multiple barrel columns consisting of 50,000 leaky integrate-and-fire neurons and 50 million synaptic connections. To argue for the success of the hardware, we reproduce in the model known thalamocortical response transformations, balanced dynamics of excitation and inhibition, and a spatiotemporal spread of activity though the superficial cortical layers. We also use SpiNNaker to run parameter-sweeping simulations to explore model parameters and multi-trial simulations to find average activities. In doing so, we hope to demonstrate significant progress toward time- and energy-tractable simulations of entire cortical areas on the million-processor SpiNNaker machines in development.

## 2. Materials and methods

### 2.1. SpiNNaker

A SpiNNaker chip contains eighteen processors, each responsible for computing the dynamics of up to 1000 leaky integrate-and-fire neurons and their afferent synapses. A single SpiNNaker board comprises 48 such chips connected by a programmable communications network. So, when a processor generates a spike for some presynaptic neuron, a network of packet-switched routers conveys that information along virtual axons to every processor on which postsynaptic neurons reside. Connectors on each edge of the network facilitate seamless tiling of boards into multi-board machines, without the communication bottlenecks suffered by, for example, GPU clusters communicating over PCI busses. Furber et al. (2013) give a broad overview of the hardware, and Sharp et al. (2012) demonstrate its extreme energy efficiency.

Each SpiNNaker processor executes a custom run-time kernel to schedule and despatch simulation *tasks*, such as membrane-potential evaluations and synaptic-current computations. The kernel executes all *tasks* in response to corresponding *events* generated in the hardware: processors compute membrane potentials every millisecond in response to timer events generated by their internal clocks; they compute synaptic currents in response to packet events that occur when the router delivers a spike to the processor. Sharp et al. (2011b) describe this level of software in greater detail.

Users of SpiNNaker may design simulations on a desktop computer, via the PyNN package for Python (Davison et al., 2009). PyNN allows users to build networks as *populations* of homogeneous neurons and *projections* of synapses, with models for each drawn from a standard library. The PyNN programming interface itself is essentially *declarative*, in that it provides a way for users to specify networks to be simulated; an implementation of the PyNN interface for a particular simulator, such as SpiNNaker, then reads these specifications and drives the simulator accordingly. However, since PyNN is a Python library, programmers may use all of the usual *imperative* and *object oriented* features of Python to build, simulate and analyze models; a program, for example, may contain loops to run repeated trials, conditional statements to guide parameter searches, or object orientation to encapsulate substructures in models. PyNN ultimately serves as an abstraction from the simulator, and does so for three reasons: to hide the great complexity of the underlying simulation technology; to ensure that models are portable between simulators; and, taking advantage of the prior feature, to verify the correctness of simulators against one another. Galluppi et al. (2012) provide further information on the implementation of PyNN for SpiNNaker, and Sharp and Furber (2013) use PyNN to demonstrate the peak performance of SpiNNaker and show that the simulator produces correct results with respect to Brian and NEST.

Although SpiNNaker is able to simulate arbitrary models, in this work we use the leaky integrate-and-fire neuron because it allows us to analytically determine desirable parameters, as discussed below. Membrane-potential dynamics for the model are given by

(1) $$\tau_{m}\frac{dV}{dt}=E_{L}-V+R_{m}I$$

where τ_*m*_ is the membrane time-constant, *E*_*L*_ is the equilibrium potential, *R*_*m*_ is the membrane resistance, and *I* is the synaptic current. We use a synapse model with first-order linear kinetics, such that the current from each set of synapses with common time-constants is given by

(2) $$\tau_{s}\frac{dI}{dt}=-I+\sum_{i\;=\;0}^{n}w_{i}\sum_{j\;=\;0}^{m_{i}}\delta (t-t_{ij})$$

where *n* is the number of synapses in the set, *m*_*i*_ is the number of spikes received on the *i*th synapse, τ_*s*_ is the synaptic-current time-constant, *w* is the weight of the synapse, δ(*x*) is the discrete-time Dirac delta function that returns 1 when *x* = 0 and 0 otherwise, and *t*_*ij*_ is the time of the *j*th spike onto the *i*th synapse. Sharp et al. (2011b) describe the methodology of simulating this model in more detail.

### 2.2. Modeling parameters

The average rat barrel column contains 18,000 neurons in a tangential area of 0.12 mm squared and a depth of 1.84 mm (Meyer et al., 2010a). The literature provides data on the sizes of constituent populations, the probability of projections and the physiology of both. Table 1 lists population sizes for the thalamus and the cortex, which were found by Oberlaender et al. (2011) and Meyer et al. (2010a) using automated counts of NeuN-positive cells in slices and the assumption that 15% of cortical neurons are inhibitory. Table 2 presents physiological properties of the model neurons, according to results of paired intracellular recordings performed by Lefort et al. (2009); synaptic-current time constants were taken from Kyriazi and Simons (1993) and Sun et al. (2006). Lefort et al. (2009), Avermann et al. (2012), Sun et al. (2006), and Meyer et al. (2010b) report on the connectivity patterns between cell types in the barrel.

**Table 1:** **Neurons per barrel column (Meyer et al., 2010a; Oberlaender et al., 2011)**.

**Population**	**Exci**.	**Inhi**.
Layer 2/3	4507	795
Layer 4	3471	613
Thalamus	285	–

**Table 2:** **Neuron-model parameters for each layer of the barrel column (Lefort et al., 2009)**.

**Parameter**	**Unit**	**L2/3E**	**L4E**
*E*_*L*_	mV	−72	−66
*V*_reset_	mV	−72	−66
*V*_Θ_	mV	−40	−40
τ_*m*_	ms	30	35
*R*_*m*_	MΩ	190	300
τ_*se*_	ms	5	5
τ_*si*_	ms	15	15
Refrac.	ms	10	10

Although the membrane properties of excitatory and inhibitory neurons are known to be different, we used identical parameters for both types of cell to simplify the analytical determination of synaptic weights.

**Figure 1:**
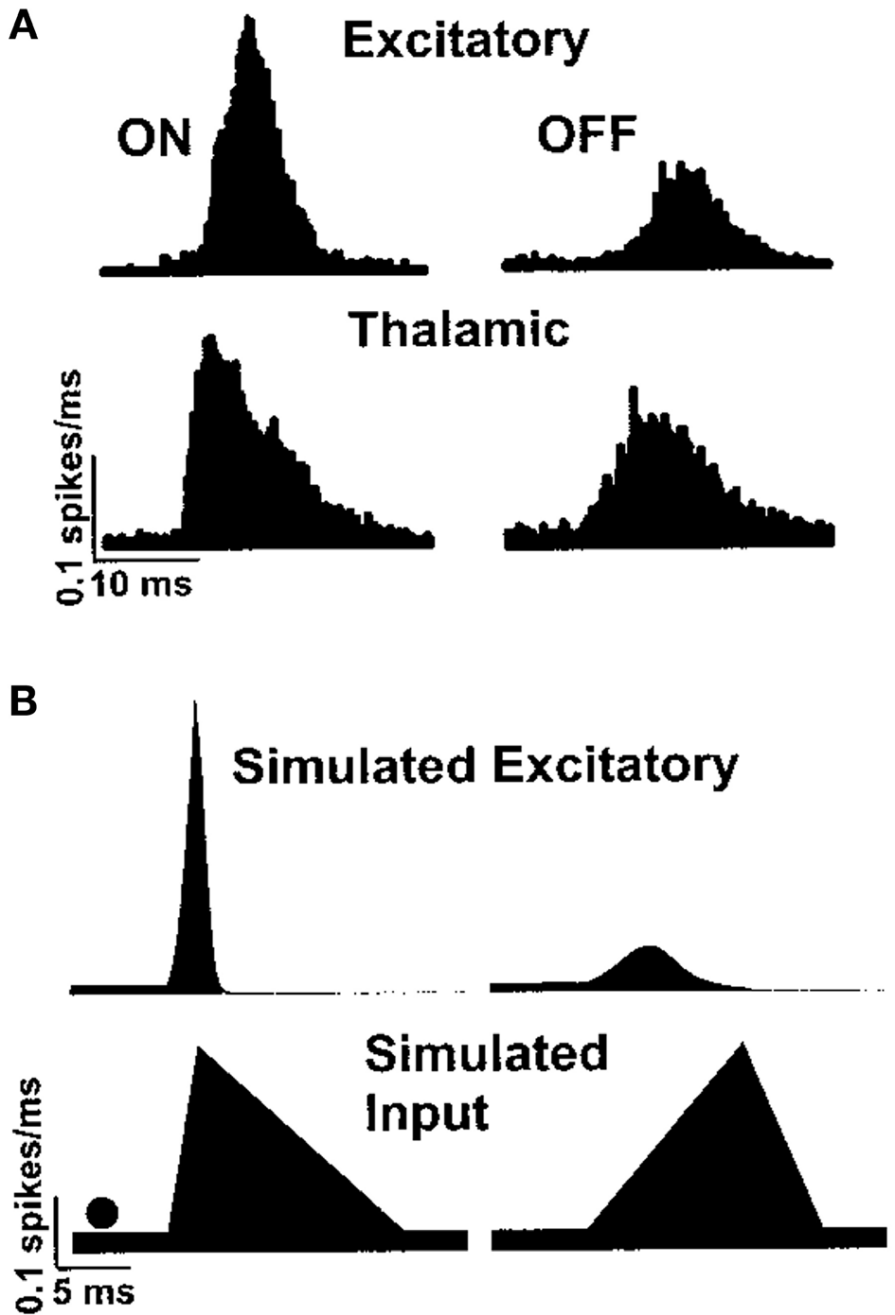
**Peristimulus time histogram of excitatory spiking in the barrel cortex in response to whisker deflection onset and offset, as shown by Pinto et al. (2003)**. Panel **(A)** shows responses recorded *in vivo* and panel **(B)** shows a simulated reproduction in a simple dynamical model.

The function of the whisker barrel has been investigated by electrophysiological recording *in vivo* from neurons in a particular barrel column while applying a mechanical “ramp and hold” stimulus to the corresponding *principal* whisker of that barrel. By comparing responses in the thalamus to those in the cortex, Simons and Carvell (1989) showed that the whisker barrel exhibits four response transformations on thalamic input, namely: thalamic neurons have greater levels of spontaneous spiking than cortical excitatory neurons; cortical excitatory neurons respond to deflection of an adjacent whisker, unlike thalamic neurons; the response of excitatory neurons to principal-whisker deflection is suppressed if it is immediately preceded by deflection of an adjacent whisker; and cortical excitatory neurons respond with different numbers of spikes to the onset and offset of whisker deflection, whereas thalamic neurons do not. Pinto et al. (2003) hypothesized that the latter transformation is a result of the rate of onset of thalamic stimuli, and proceeded to reproduce such behavior in a dynamical model of the whisker barrel, as shown in Figure 1.

### 2.3. Modeling principles

In a model composed of excitatory and inhibitory populations with recurrent projections, an important factor in spiking activity is the density and weight of synaptic connections. Should the number and strength of excitatory synapses outweigh those of inhibitory synapses, for example, recurrent excitation will dominate and the network will become hyperactive. Should inhibition dominate, the network will become inactive. An important principle of cortical function seems to be that excitation and inhibition are in balance (Shadlen and Newsome, 1994; van Vreeswijk and Sompolinsky, 1996). Brunel (2000) derives quantitative conditions for such balance in networks of leaky integrate-and-fire neurons. He considers a model comprising *N*_*e*_ excitatory and *N*_*i*_ inhibitory neurons, driven by external input, where each neuron receives a synapse from any other neuron with equal probability *p*. The number of synapses formed within the network is *p*(*N*_*e*_ + *N*_*i*_)^2^ so the ratio of excitatory to inhibitory synapses is *N*_*e*_/*N*_*i*_. Given some arbitrary excitatory synaptic weight *w*_*e*_, a significant relationship to the inhibitory weight *w*_*i*_ is described by

(3) $$w_{i}=bw_{e}\frac{N_{e}}{N_{i}}$$

where *b* is a balance coefficient. Brunel observes the firing rate of the model as a function of *b* and shows that excitation drives hyperactivity when *b* is less than one, that inhibition causes hypoactivity when *b* is greater than one, and that the two forces counterbalance when *b* is exactly one. Thus, the Brunel equation is useful for analytically determining the parameters of a stable model of the whisker barrel, since the barrel has been described as two populations, one excitatory and one inhibitory, driven by a sole thalamic input (Kyriazi and Simons, 1993; Pinto et al., 2003). Kyriazi and Simons use varying synaptic-current time constants for excitatory and inhibitory synapses in their model, and we wish to reproduce this feature. The charge imparted by a single postsynaptic current of the type we model with amplitude *w* and time constant τ_*s*_ is

(4) $$\int_{0}^{\infty }\text{​}w\exp\text{​}\left(-t/\tau_{s}\right)dt=w\tau_{s}$$

So we can extend the Brunel equation to consider the balance between postsynaptic currents thus:

(5) $$w_{i}\tau_{si}=bw_{e}\tau_{se}\frac{N_{e}}{N_{i}}$$

## 3. Results

We conducted three sets of simulations to demonstrate various capabilities of SpiNNaker and to test that the SpiNNaker barrel-model performed correctly. To this end, we firstly ran parameter-sweeping simulations to verify that the model satisfies the relationship between excitatory and inhibitory balance described analytically by Brunel; secondly, we reproduced the thalamocortical response transformations observed and simulated, respectively, by Simons and Carvell (1989) and Kyriazi and Simons (1993); and finally we simulated a chain of five barrel columns in parallel to show the scale of models feasible on SpiNNaker.

### 3.1. Parameter sweeping

We first simulated one barrel, consisting of dual populations of excitatory and inhibitory neurons, driven by a common thalamic input. We simulated thalamic neurons as Poisson spike trains and cortical neurons with the leaky integrate-and-fire model, and set population sizes and neuronal biophysical parameters according to Tables 1, 2. We connected thalamic neurons to cortical ones with a probability of 0.25 and chose thalamocortical weights to be the minimum needed to elicit firing in a model without intracortical projections under stimulus from 6 Hz Poissonion thalamic firing (Bruno and Sakmann, 2006). We set all intracortical projection probabilities to 0.1, fixed the excitatory synaptic weight at 0.1 nA, and determined the inhibitory weight by Equation (5), with *b* varying from 0.1 to 10 in successive trials. In each trial, we instantiated and loaded the model once and then simulated it 10 times with varying seeds for the Poisson spike source; we recorded the average excitatory firing rate during each one-second simulation, and took the mean and standard deviation of these 10 numbers as ultimate result of the trial. Using PyNN, we specified and executed these simulations with a single Python program: a simulation function specified a network, triggered a simulation and retrieved results (all the mechanics of which were hidden behind the PyNN interface) and a controller function called the simulation function in a loop over varying *b*-values and analyzed the collated results.

**Figure 2:**
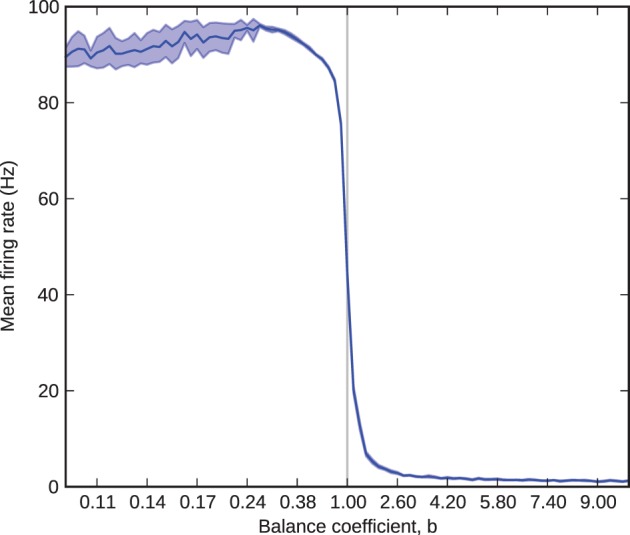
**Barrel firing rate as a function of the balance coefficient, b**. The solid blue line represents the 10-trial mean and the shaded area represents one standard deviation over the same 10 trials.

Figure 2 shows mean and standard deviation of excitatory firing rate in the barrel as a function of the balance coefficient. The results follow the expected curve closely: as *b* sweeps from less than to greater than one, the firing rate transitions from near-maximum to near-minimum through a tight sigmoid curve centered upon *b* = 1. The exception to the expected results is for values of *b* less than 0.25. Here, at firing rates close to 100 Hz each processor (performance must be considered on a per-processor basis, because network performance is inexhaustible relatively) receives approximately 400 spikes per millisecond, each of which innervates on average 10% of the 256 neurons simulated on the processor. This rate of synaptic events (afferent spikes multiplied by innervated synapses) exceeds peak throughput (Sharp and Furber, 2013) so some spikes are lost. Consequently, for values of *b* less than 0.25 the standard deviation grows as a result of trial-to-trial performance variability and the mean falls correspondingly because the variance causes only the loss, not gain, of spikes. The problem of modeling high firing rates in monolithic, recurrently connected populations on SpiNNaker can be mitigated by splitting them into smaller subpopulations, relaxing the real-time performance schedule, or modeling fewer neurons, and therefore fewer synapses, per processor.

**Figure 3:**
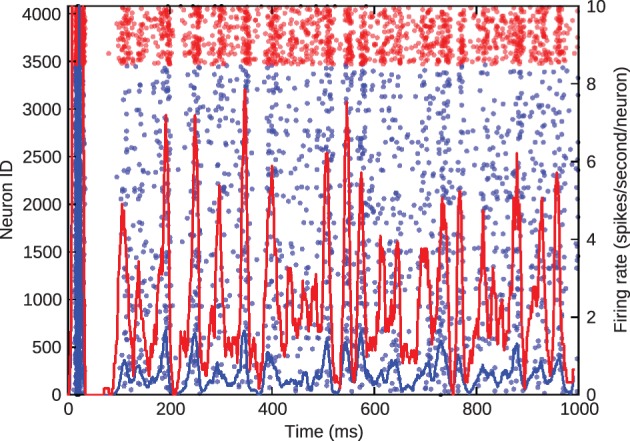
**Asynchronous, irregular spiking in the barrel model**. Blue represents excitatory neurons and red represents inhibitory neurons. The left *y*-axis gives the ID of the spiking neurons in the scatter plot. The right *y*-axis gives the average firing rate of the excitatory and inhibitory populations, denoted by the solid lines.

Figure 3, where blue denotes the activity of excitatory cells and red the inhibitory activity, shows the barrel firing asynchronously at a realistic rate of approximately 1 Hz when just the inhibitory-to-excitatory weights are multiplied by 1.5 to control excitatory firing rate.

### 3.2. Thalamocortical response transformations

**Figure 4:**
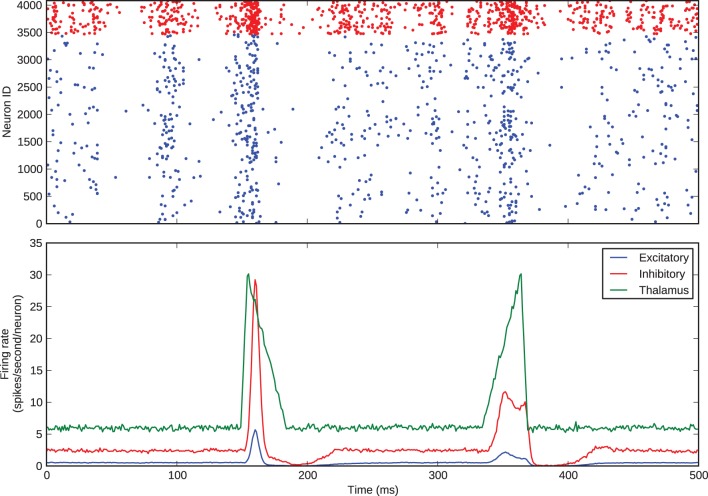
**Thalamocortical response transformation in the whisker barrel**. The top panel shows the spikes from a single trial; the bottom panel shows average firing rates across all 25 stimulus presentations to all 10 model instances.

We simulated this same barrel, with the excitatory-inhibitory balance used to generate Figure 3, to examine thalamocortical response transformations in the model. The two transformations we considered were the lower firing rate of layer 4 excitatory neurons with respect to thalamic neurons and the differential response of excitatory neurons to onset and offset stimuli. Bruno and Sakmann (2006) show that, under the experimental conditions of the data that we are considering, thalamic neurons spontaneously spike at an average rate of 6 Hz, so we set the model thalamic cells to fire spontaneous Poisson trains at this rate. As discussed above and shown in Figure 1, Pinto et al. (2003) show that the thalamic responses to whisker-deflection onset and offset differ principally in terms of onset rate, and we varied the firing rate of the thalamic spike trains to mimic this: onset stimuli triggered a stimulus *triangle* rising from 6 to 30 Hz in 5 ms and then decaying back to 6 Hz in 30 ms; offset stimuli triggered a stimulus triangle of equal amplitude and opposite rise and decay times. These patterns of thalamic stimuli can be seen in the green trace in the lower panel of Figure 4. In our simulations, each stimulus battery comprised one whisker deflection onset and one offset separated by 150 ms, preceded by a 500-ms rest period. We instantiated 10 barrel models and delivered 25 stimulus batteries to each. Again, the function and loop constructs inherent in Python made the specification and execution of multiple trials simple.

Figure 4 shows the barrel spikes resulting from one stimulus battery and the mean firing rates across all of the 25 batteries to all 10 model instances. For clarity the stimulus battery is centered in the figure. The barrel model clearly reproduces the two response transformations discussed by Simons and Carvel and Pinto et al.: firstly, excitatory neurons fire asynchronously at a mean rate much lower than that of thalamic neurons; and secondly, onset stimuli elicit greater firing rates in excitatory neurons than offsets.

### 3.3. Multiple barrel columns

Whereas recurrent connectivity in layer 4 is almost entirely confined to each individual barrel, there are more extensive interbarrel connections in the supragranular layers, which are important for the lateral spread of neural activity across the cortex (Petersen and Diamond, 2000; Civillico and Contreras, 2006). Investigating this spread of activity requires a large-scale multi-barrel model.

**Figure 5:**
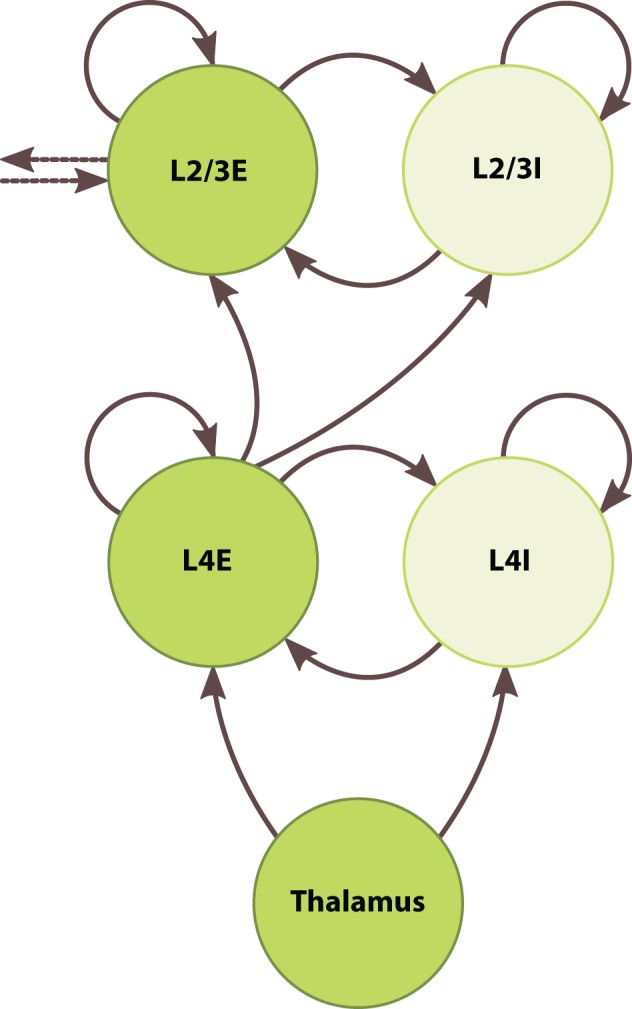
**Architecture of the barrel-column model**. Circles represent discrete populations. Solid and dotted arrows represent intra- and interbarrel synaptic projections, respectively.

To develop the large-scale modeling capabilities of SpiNNaker, we built a chain of five inter-connected barrel columns each consisting of a granular and a superficial layer. Figure 5 depicts one such column. Each layer contained one excitatory and one inhibitory population, which were connected recurrently and to one another. The thalamus fed simulated whisker signals to both populations of the granular layer, and the excitatory granular neurons relayed signals to the supragranular layers. Supragranular populations formed lateral projections with their immediate neighbors in the chain. We did not address edge effects at the ends of the chain because we only sought to show a unidirectional propagation of activity through the supragranular layers.

We set supragranular population sizes according to Table 1. As with the granular layer, we set all intracortical projection probabilities in the supragranular layer to 0.1, fixed the excitatory synaptic weight at 0.1 nA, and determined the inhibitory weight by Equation (5), adding 50% to the inhibitory-to-excitatory weight to keep firing rates in a biologically plausible range. Between layers, we tuned projection parameters to elicit a baseline firing rate of around 1 Hz: we set the projection probability from the excitatory granular neurons to both supragranular populations to 0.1, consistent with the other intracortical projections, and set the synaptic weight of both projections to 0.2 nA. Between columns, we formed lateral projections between excitatory populations of the supragranular layers, with probability 0.1 and synaptic weight of 0.1 nA. We specified columns as Python objects with PyNN populations and projections as their attributes, again taking advantage of basic Python features to simplify the simulation of these complex networks. We instantiated five columns, forming a model of 5 · 10^4^ neurons and 5 · 10^7^ synapses to be simulated across 200 processors on 13 chips.

**Figure 6:**
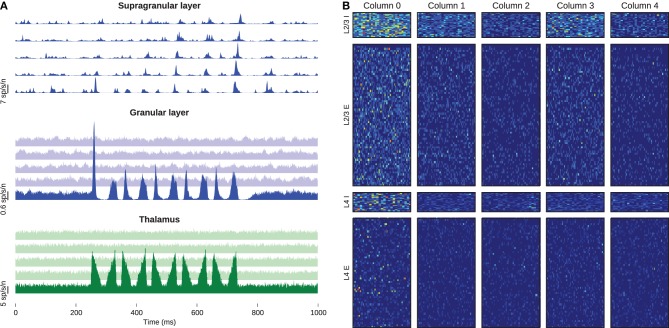
**Activity of the barrel-column chain**. **(A)** Excitatory peristimulus spike counts of whole populations, showing signal propagation through the chain of barrel columns. The top, middle, and bottom panels represent the supragranular, granular and thalamic populations, respectively, and the five traces in each panel from bottom to top represent the five columns from left to right. Note the varying *y*-scale bars on each panel. **(B)** Post-stimulus spike-counts of every cell in the simulation. Each column of panels belongs to one barrel column, and each row corresponds to the populations of the barrel. The heat-map colors in each row are normalized to the highest spike count of that row.

We defined one stimulus battery as five repetitions at 10 Hz of the whisker-deflection onset and offset used above, preceded by a 500-ms rest period. We delivered five batteries to the leftmost column while supplying the others with only baseline stimulus. Figure 6A shows the peristimulus time histogram in spikes per second per neuron, averaged across all stimulus-presentations, again centered upon the stimulus battery. The thalamic stimulus activates barreloid 0, which in turn excites neurons in the corresponding barrel. Neurons in the other barreloids and barrels receive no external drive and hence show no evoked response. The activated granular layer in barrel 0 excites the corresponding supragranular layer, from which the firing apparently propagates along the chain of columns. Figure 6B shows the spike counts of every neuron, summed across 25-ms windows following the onset of each of the 25 whisker deflections. Excitatory cells in layer 4 represent the stimulus with sparse firing into layer 2/3, which in turn shows a much denser response in the proximal column and sparser responses in the distal neighbors. These models, comprising some 50 million synapses, are to-date the largest recurrent networks simulated in real-time.

## 4. Discussion

Simulating neural circuits is a promising approach to improving our understanding of brain function. However, the nervous system is enormously complex in structure and simulating even small neural circuits is still a difficult problem. Evaluating the membrane potentials of many thousand of model neurons requires great computational parallelism, and communicating action potentials between these neurons requires programmable communications.

General-purpose supercomputers do meet these requirements. Markram (2006) describes the hardware and software architecture of the Blue Brain Project, which intends to use 2^17^ processors in an IBM Blue Gene/L computer to simulate 10^4^ cortical neurons and their 5·10^7^ synapses in great physiological detail. Ananthanarayanan et al. (2009) use 2^16^ processors in an IBM Blue Gene/P machine to simulate 10^9^ simpler, single-compartment (Izhikevich, 2003) neurons and 10^13^ synapses, arguing that this portends full-scale real-time simulations of the human cortex. However, the power requirements of conventional supercomputers render this vision impossible; Sharp et al. (2012) estimate that the simulations proposed by Ananthanarayanan et al. would draw approximately 10 gigawatts.

Graphics processing units (GPUs) contain tens or hundreds of arithmetic units that can execute a single instruction stream on many data elements simultaneously. Many authors have exploited this property of GPUs to simulate up to tens of thousands of neurons in parallel (Nageswaran et al., 2009; Bhuiyan et al., 2010; Fidjeland and Shanahan, 2010; Han and Taha, 2010; Pallipuram et al., 2011; Nere et al., 2012). More recently, Beyeler et al. (2014) and Minkovich et al. (2014) have presented GPU simulations of hundreds of thousands of neurons and tens of millions of synapses. Beyeler et al. use an off-the-shelf GPU to simulate 40 million synapses in real-time, and Minkovich et al. approach the significant and previously unaddressed problem of multi-GPU simulations, thereby promising very large-scale simulations across many processors. However, SpiNNaker retains some advantages over current GPU studies. As we tend toward brain-scale simulations power-efficiency becomes increasingly important, and Sharp et al. (2012) have shown that SpiNNaker outperforms conventional architectures in this regard, whereas high-performance GPUs tend to be very power-hungry. SpiNNaker also outperforms conventional multiprocessors for neural-circuit simulations when compared on a basis of equal multiply-accumulate operations per second (Sharp and Furber, 2013); the “like-for-like” performance of GPUs remains unclear, however, as GPU speedups are often reported on the basis of comparison between disparate architectures (Lee et al., 2010). Finally, the communications architecture of SpiNNaker is designed to handle the dense, highly divergent synaptic connections observed in the cortex (Sharp and Furber, 2013) but such connections still present a challenge for GPU communications, as suggested by the low synaptic densities achieved by Beyeler et al. and Minkovich et al. about 250 and 100 per neuron, respectively.

The BrainScaleS architecture presents a promising solution to these problems (Schemmel et al., 2010). BrainScaleS intends to enable the kind of parameter-sweeping, scalable, multi-trial simulations demonstrated here using a large-scale implementation of the established, exceptionally energy-efficient practice (Mead, 1989) of simulating neurons using the subthreshold dynamics of transistors. Furthermore, BrainScaleS aims to solve the existing problem of spike-communications in analog circuits using an auxiliary, digital packet-switched network, similar to SpiNNaker's. However, the project still faces the problem that analog circuits are difficult to tune for particular behaviors (Brüderle et al., 2011). This problem grows with the number of neurons simulated, so that it may difficult to build a population with homogeneous, or particular distributions of, parameters.

SpiNNaker is a digital computer architecture that emulates the structure and function of neural computation, using very many low-power processors and an interprocessor communication mechanism inspired by axonal arbors, to efficiently simulate neural tissue. SpiNNaker differs from conventional supercomputers in that the processors eschew high clock-speeds and floating-point units in favor of energy efficiency, the communications infrastructure contains little hardware specifically for system control and debugging, and there is little processor time and memory available for monitoring and debugging processes. This does present some challenges to using the architecture. Firstly, we must compute all neuron and synapse states in fixed, rather than floating, point arithmetic; this increases program complexity a little, but does not have any significant effect on the accuracy of the simulator (Sharp and Furber, 2013). Secondly, we must load data structures for simulation to each processor over the packet-switched network, which is costly (Sharp et al., 2011a) although the loading time may be significantly shortened by compression methods that we are currently developing. Finally, we must debug programmes with little information relative to the scale of the system. This last problem is likely common to all massively-parallel architectures, but it remains the most significant outstanding challenge for SpiNNaker.

Nevertheless, this paper demonstrates the success of prototype, thousand-processor SpiNNaker hardware using a software stack orientated to the interests of computational neuroscientists. In these simulations, we have demonstrated that a complex, massively-parallel machine can be used to rapidly simulate neural circuits using a simple declarative library for Python. We believe SpiNNaker may hence contribute to research in computation neuroscience in three ways.

Firstly, parameter-sweeping experiments are useful because analytical descriptions of complex network behaviors are rare. To determine, for example, the excitatory-inhibitory current balance of networks more complex than Brunel considers, researchers may simulate models at each point in the parameter space. This approach requires either great sequential performance or great parallelism. SpiNNaker offers both, in that it simulates in real-time and may run many model instances in parallel. The coupling of declarative PyNN with imperative Python allows researchers to specify, in a single concise program, a model to be simulated *and* a procedure to follow for multiple parameters, trials and model instances.

Secondly, multiple runs of simulations are necessary to establish the statistical significance of modeling results. For the same reasons as above, and using the same methods, SpiNNaker is useful in such procedures.

Finally, certain research questions may be answered by large-scale simulations. In order to explain the effect of attentional signals on stimulus-response in the visual cortex, Wagatsuma et al. (2011) model columns in the visual cortex containing some 80,000 neurons. Phoka et al. (2012) model a smaller circuit comprising a single barrel column, but with computationally expensive STDP, to examine the effect of whisker stimuli on the synaptic state of the network. To explore the high-level computational functions of cooperating neural systems, Eliasmith et al. (2012) present a model encompassing more than two million neurons in brain areas from visual input through processing to motor output. In all cases, simulations may be accelerated by specialized computing hardware. In support of this argument, we have demonstrated here that SpiNNaker can efficiently simulate some part of the rodent barrel cortex. We modeled five barrel columns using 200 processors of a prototype SpiNNaker board and a software stack designed to simplify the use of the machine. We created a Python class to represent a single barrel of PyNN populations and projections, and then created instances of this class to form a model of around 50,000 neurons and 50 million synapses. These demonstrations are a significant step toward tractable simulations of entire cortical areas on the million-processor SpiNNaker machines in development.

### Conflict of interest statement

The authors declare that the research was conducted in the absence of any commercial or financial relationships that could be construed as a potential conflict of interest.
